# Recording mobile EEG in an outdoor environment reveals cognitive-motor interference dependent on movement complexity

**DOI:** 10.1038/s41598-019-49503-4

**Published:** 2019-09-11

**Authors:** Julian Elias Reiser, Edmund Wascher, Stefan Arnau

**Affiliations:** Leibniz Research Centre for Working Environment and Human Factors (IfADo) at TU Dortmund, Dortmund, Germany

**Keywords:** Cognitive control, Psychology

## Abstract

Oftentimes we find ourselves in situations in which we need to perform concurrent motor and cognitive tasks like simple locomotion while being cognitively involved. In the present study, we investigated in how far cognitive and motor functioning interfere in an outdoor environment. Our participants performed an auditory oddball task while concurrently completing various motor tasks on the outside premises of our institute. Beside behavioural responses and subjective workload ratings, we also analysed electrophysiological data recorded with a 30-channel mobile EEG montage. We observed an increase of subjective workload and decrease of performance with increasing movement complexity. Accordingly, we also found a decrease in the parietal P3 amplitude as well as in frontal midline Theta power with higher motor load. These results indicate that an increased movement complexity imposes a higher workload to the cognitive system, which, in turn, effectively reduces the availability of cognitive resources for the cognitive task. Overall this experiment demonstrates the feasibility of transferring classical paradigms of cognitive research to real-world settings. The findings support the notion of shared resources for motor and cognitive functions by demonstrating distinct modulations of correlates of cognitive processes across different motor tasks.

## Introduction

Movement and higher cognitive processes go hand in hand in highly developed organisms. Even more so, performing a cognitive task while in motion constitutes a great part of our daily life routine. The mutual interdependence of concurrent movement and cognitive activities, however, is not yet fully understood. De Sanctis^1^ argued that most cognitive experiments are currently using a “minimalistic behavioural approach, reducing behaviour in response to task relevant stimuli to simple button presses”, thus favouring internal over ecological validity. In the real world, on the other hand, humans must locate their body and their actions in the environment in order to react to certain events^2^. For this reason, cognition in motion accompanied by natural behaviour can only be examined in sufficiently realistic environments.

The importance of a step towards ecological validity in cognitive psychology has been debated for decades^3^. Moving out of the laboratory into natural living and working environments may allow for valuable insights into the nature of cognitive processing^4,5^. Recent research supports this notion by permitting participants to exhibit realistic behaviour^6–8^ and natural responses^9,10^. Several studies demonstrated significant differences between laboratory and realistic settings with respect to performance as well as with respect to electrophysiological measures. For example, a decreased cognitive performance accompanied by an altered reflection of the corresponding processes in the EEG was reported for cycling freely outdoors compared to cycling indoors on an exercise bike^11,12^. A similar result pattern could be observed for different movement conditions. Debener *et al*.^13^ demonstrated the feasibility of outdoor dual-task studies involving a walking and a cognitive task. The participants showed a decreased cognitive performance when walking outdoors on the campus as compared to sitting indoors. The elctrophysiological results of Debener *et al*.^13^ also clearly show that correlates of cognitive processing in the EEG may look different in real world settings as compared to laboratory studies.

This altered representation of cognitive processes in the EEG in real world settings might be due to various reasons. Firstly, the sensory input is vastly increased in an outdoor or workspace setting compared to a laboratory environment. Secondly, the embedding of a task in a sensorially rich environment may increase task complexity per se. Both sources of variance, as well as their interaction, most likely alter task processing by directly affecting attentional processes and the distribution of cognitive resources in comparison to simplified lab settings or to virtually simulated experiments^14^. Building the foundation for real-world investigations, several studies demonstrated that a higher ecological validity does not necessarily compromise internal validity. This was demonstrated by several dual-task experiments, for example involving manual work^15^ or driving a real car^16^. Studies investigating concurrent performance in a motor and a cognitive task reported decrements for both the cognitive as well as the motor domain^13,17,18^. Decrements in either the cognitive or the motor domain due to the simultaneous execution of two tasks are termed interference^18^.

The question why motor tasks lead to a cognitive performance decrement and vice versa is still discussed^18,19^. A traditional view is that highly automated processes – like ordinary locomotion – do not take up attentional resources^20^. A common explanation for dual-task interference involving locomotion, however, is the scarcity of attentional resources^21,22^. Relating to Baddeley^23,24^, the distribution of attentional resources during dual-tasking is guided by executive functions which are related to higher cognitive processes. In their meta-analysis, Al-Yahya *et al*.^25^ showed that many measures of gait, especially walking speed, are negatively affected by a secondary cognitive task. They conclude, that both motor and cognitive processes share a common resource. Recent brain imaging studies revealed that areas related to executive functions are activated mainly in the prefrontal cortex area while or in preparation to walking, which endorses the shared resources theory^26–28^. This holds also true during actual cognitive-motor dual-task walking^29,30^. Accordingly, when paired with a cognitive task, gait and coordinated movement have been shown to induce an additional need for processing resources^1,31^. This dual-task interference may result in diminished performance and could therefore lead to erroneous behaviour^32,33^. Overall, it seems that, although highly automated, ordinary locomotion uses the same attentional resources as cognitive processing.

As outlined above, recent research clearly demonstrated, that sensory and behavioural complexity is substantially increased in real world compared to laboratory settings. This affects the task-related executive control functioning, especially if movement is involved^9^. Even ordinary movement sequences like walking, cycling, and balancing may influence cognitive processing^1,11,12,14,31^. However, no research has been concerned with the cognitive underpinnings of gait movement complexity while performing a cognitive-motor dual-task in a non-laboratory bound investigation. Focusing on gait, we wanted to investigate, how the complexity of different movement conditions influences attentional resources in a semi-standardized outdoor setting. In the present study, the participants had to either stand, walk, or complete an obstacle course while performing a simple, auditory stimulus detection task on the lawn of the institute’s outside premises. We used a modified version of the auditory oddball paradigm by Debener *et al*.^13^ which has been proven to be suitable for investigating outdoor cognitive processing. In order to gain further insights into the interplay of motor complexity and the cognitive task, we introduced two target probability levels in the oddball task: high target probability (HTP) with 35% target stimuli vs. low target probability (LTP) with 20% target stimuli. Since the manipulation of target probability showed effects on ERP amplitudes in earlier laboratory-based oddball studies^34–36^, we aimed to use this concept as a proof-of-concept factor in our outdoor experiment. By inclusion of target probability, we tried to answer the underlying question, whether we can find the same differences in standard and target ERP amplitudes which were found before in lab environments. We were also curious which effects the interaction between movement and target probability had on attentional resources. Overall, participants completed six conditions consisting of one of each factorial combination in quasi-randomized order. Besides reaction time measures and the number of target trial omissions, we assessed the subjective workload of the participants by using the NASA-TLX questionnaire after every task block^37^. Furthermore, we applied mobile EEG in order to measure electrophysiological correlates of task-related cognitive processes.

Over the last few years, electrophysiological recording equipment has been miniaturized to the point that one can measure EEG wirelessly over the period of several hours, thus enabling us to record neurophysiological data outside of the lab. In the present study, we investigated frontal midline Theta power as well as the frontal N2 and parietal P2, N2, and P3 event-related potential (ERP) components. The fronto-central N2 and the parieto-temporal P2, N2, and P3 were shown to vary with resource allocation^38^. The P2 component, the second positive deflection after stimulus presentation, was found to resemble early attentional processes in auditory and visual tasks^39,40^. The N2 component, the second negative deflection after stimulus presentation, is thought to reflect conflicts in task response execution or inhibitory processes as well as general executive control mechanisms^41,42^. The P3 component, the third positive deflection after stimulus onset, has been associated with stimulus updating and categorization processes^43^ and appears to be negatively correlated with task difficulty^44^. For all components, a high task difficulty in a dual-task setting was linked to diminished amplitudes^38^. The analysis of event-related spectral power offers an additional opportunity to assess cognitive processing in the EEG^45^. Spectral power in the Theta range (4–7 Hz) has been associated with the execution of executive control, probably reflecting communication between areas featuring a fronto-central hub^46,47^. Therefore, a comparatively strong fronto-central Theta activation, being strongly related to the invested mental effort or resources, is typically observed in experimental conditions with increased cognitive control demands^46,48–50^.

As an error in the motor task may lead to falling and possible injuries, we expected that the participants would prioritize the motor task, leading to higher subjective workload ratings for more complex movement complexity conditions. According to the literature discussed above, we therefore hypothesize that increased movement complexity will also lead to a decrease in the amount of cognitive resources that may be expended for the cognitive task. As a consequence, fewer attentional resources should be directed towards the cognitive task when the motor task demands are high. Furthermore, we expect a decreased behavioural performance in high compared to low movement complexity conditions due to dual-task interference. The same logic applies to the electrophysiological measures, as we analysed the EEG time-locked to the onset of the stimuli of the cognitive task. The amplitudes of stimulus-locked P2, N2 and P3 components as well as frontal Theta power should be decreased with higher movement complexity, as fewer attentional resources are available to the cognitive task. According to recent findings, target probability should affect response times. As a proof-of-concept, we correspondingly expect slower response times with lower target probability^51^. Also P2, N2 and P3 amplitudes within targets should be diminished as target probability is increased^34–36^, as should Theta power^52^.

## Results

### NASA-TLX

**Figure 1:**
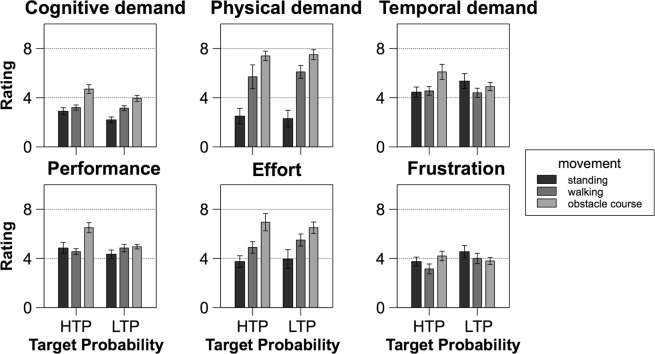
Means of subjective NASA-TLX ratings for all dimensions seperately. Error bars depict the standard error. HTP: high target probability (35%), LTP: low target probability (20%).

All dimensions on the NASA-TLX are poled and analysed with low rating values reflecting low subjective workload. Figure 1 illustrates the average ratings and the standard error of all subscales of the NASA-TLX for each experimental condition combination. All results of the separate ANOVAs are shown in Table 1.

**Table 1:** Results for repeated-measures ANOVAs for the factors movement complexity, target probability and their interaction term regarding every NASA-TLX dimension. C: Cognitive demand, Ph: Physical demand, T: Time demand, Pe: Performance, E: Effort, F: Frustration, TP: Target probability.

Variable	Move			TP			Interaction		
	F	p	${{\boldsymbol{\eta }}}_{{\boldsymbol{p}}}^{2}$	F	p	${{\boldsymbol{\eta }}}_{{\boldsymbol{p}}}^{2}$	F	p	${{\boldsymbol{\eta }}}_{{\boldsymbol{p}}}^{2}$
C	*F*_2,38_ = 17.70	<0.001	0.45	*F*_1,19_ = 6.73	0.018	0.26	*F*_2,38_ = 2.05	0.143	0.10
Ph	*F*_2,38_ = 25.44	<0.001	0.57	*F*_1,19_ = 0.10	0.757	0.00	*F*_2,38_ = 0.53	0.591	0.03
T	*F*_2,38_ = 2.95	0.065	0.13	*F*_1,19_ = 0.21	0.654	0.01	*F*_2,38_ = 2.03	0.168	0.10
Pe	*F*_2,38_ = 6.50	0.004	0.25	*F*_1,19_ = 3.02	0.099	0.14	*F*_2,38_ = 5.47	0.018	0.22
E	*F*_2,38_ = 9.53	0.002	0.33	*F*_1,19_ = 0.07	0.792	0.00	*F*_2,38_ = 0.56	0.575	0.03
F	*F*_2,38_ = 0.89	0.373	0.04	*F*_1,19_ = 2.63	0.121	0.12	*F*_2,38_ = 1.74	0.199	0.08

#### Cognitive demand

The target probability main effect showed that participants rated high target probability (*M* = 3.6, *SD* = 1.99) as significantly more cognitively demanding (*F*_1,19_ = 6.73, *p* = 0.02, *p*_crit_ = 0.033, ${\eta }_{p}^{2}$ = 0.26) than low target probability (*M* = 3.1, *SD* = 1.64). Regarding the main factor of movement complexity, more complex movement was linked to the higher cognitive demand rating (M_stand_ = 3.85, SD_stand_ = 3.15; M_walk_ = 5.20, SD_walk_ = 2.40; M_obstacle_ = 6.73, SD_obstacle_ = 2.96; *F*_1,43,27,23_ = 15.70, *p* < 0.001, *p*_crit_ = 0.05, ${\eta }_{p}^{2}$ = 0.45). The post-hoc comparisons revealed that parcours differed significantly from standing (t_38_ = −5.52, *p* < 0.001, *p*_crit_ = 0.05) and walking (t_38_ = −3.58, *p* = 0.001, *p*_crit_ = 0.033), but means of standing and walking did not vary significantly (t_38_ = −1.94, *p* = 0.059, *p*_crit_ = 0.017).

#### Physical demand

Only the main effect of movement complexity of the physical demand dimension showed significance (*F*_1,21,23,02_ = 25.44, *p* < 0.001, *p*_crit_ = 0.05, ${\eta }_{p}^{2}$ = 0.57). The obstacle course was rated the most demanding (*M* = 7.45, *SD* = 1.97), followed by walking (*M* = 5.90, *SD* = 2.46) and standing (*M* = 2.40, *SD* = 3.19). Standing differed significantly from walking (t_38_ = −4.82, *p < *0.001, *p*_crit_ = 0.033) and parcours (t_38_ = −6.96, *p* < 0.001, *p*_crit_ = 0.05), though ratings of walking and the obstacle course did not deviate significantly after correction (t_38_ = −2.14, *p* = 0.04, *p*_crit_ = 0.017). There was neither an influence of target probability on the rating of physical workload, nor a significant interaction term.

#### Temporal demand

Concerning the temporal demand, only the main effect of movement complexity showed an acceptable effect size – but no significance (*F*_2,37,96_ = 2.95, *p* = 0.06, *p*_crit_ = 0.05, ${\eta }_{p}^{2}$ = 0.13) – with only walking and the obstacle course displaying a significant difference (t_38_ = −2.42, *p* = 0.02, *p*_crit_ = 0.05). Interestingly, ratings for the obstacle course were the highest (*M* = 5.50, *SD* = 2.53), followed by standing (*M* = 4.90, *SD* = 2.63) and then walking (*M* = 4.48, *SD* = 1.77) regarding simple means. Therefore, the hypothesized change in difficulty regarding movement complexity did not translate into higher ratings. Similar to the previous measure, neither target probability nor the interaction of both main effects revealed significant differences.

#### Performance

In terms of self-evaluated performance, the main effect of movement complexity reached significance (*F*_1,90,36,02_ = 6.50, *p* = 0.004, *p*_crit_ = 0.05, ${\eta }_{p}^{2}\,$ = 0.25), whereas the main effect of target probability did not (*F*_1,19_ = 3.02, *p* = 0.10, *p*_crit_ = 0.017, ${\eta }_{p}^{2}\,$ = 0.14). Still, target probability’s effect size indicates a certain shift in means. Estimated performance was statistically different for standing (*M* = 4.60, *SD* = 2.02) and the obstacle course (*M* = 5.73, *SD* = 1.84; t_38_ = −3.26, *p* = 0.002, *p*_crit_ = 0.05), and walking (*M* = 4.70, *SD* = 1.36) and the obstacle course (t_38_ = −2.97, *p* = 0.005, *p*_crit_ = 0.033). Participants rated their own performance to be better for low target probability (M_LTP_ = 4.72, SD_LTP_ = 1.65; M_HTP_ = 5.30, SD_HTP_ = 2.49). Performance was evaluated worse for higher movement complexity, though significant differences appeared only for standing and the obstacle course as well as walking and the obstacle course. Also, the interaction term reached significance (*F*_1,39,26,36_ = 5.47, *p* = 0.02, *p*_crit_ = 0.033, ${\eta }_{p}^{2}\,$ = 0.22). Post-hoc pairwise comparisons showed that for HTP only standing (*M* = 4.85, *SD* = 2.05) and the obstacle course (*M* = 6.50, *SD* = 1.84; t_72.93_ = −3.71, *p < *0.001, *p*_crit_ = 0.043) as well as walking (*M* = 4.55, *SD* = 1.07) and the obstacle course (t_72.93_ = −4.38, *p* < 0.001, *p*_crit_ = 0.05) were significantly different. Movement complexity manipulations did not influence the means of self-evaluated performance within LTP conditions.

#### Effort

With respect to the overall effort, only movement complexity had a significant influence on rating (*F*_1,51,28,61_ = 9.53, *p* = 0.002, *p*_crit_ = 0.05, ${\eta }_{p}^{2}\,$ = 0.33). Subjective effort was rated higher for higher complexity (M_stand_ = 3.85, SD_stand_ = 3.15; M_walk_ = 5.20, SD_walk_ = 2.40; M_obstacle_ = 6.73, SD_obstacle_ = 2.96). Post-hoc tests revealed significant differences for standing and the obstacle course (t_38_ = −4.36, *p* < 0.001, *p*_crit_ = 0.05) as well as walking and the obstacle course (t_38_ = −2.31, *p* = 0.02, *p*_crit_ = 0.033), but not between standing and walking after correction (t_38_ = −2.05, *p* = 0.05, *p*_crit_ = 0.017). Target probability and the interaction term did not affect the ratings significantly.

#### Frustration level

There were no differences regarding the subjective frustration rating, neither for both main effects, nor for the interaction term.

### Gate-related behavioural measures

Regarding the number of steps, only the main effect of movement complexity turned out to be significant (*F*_1,19_ = 15.52, *p* = 0.001, *p*_crit_ = 0.05, ${\eta }_{p}^{2}\,$ = 0.45) with a higher number of steps taken during walking (*M* = 1532.80, *SD* = 150.64) in contrast to the obstacle course(*M* = 1465, *SD* = 163.05). Target probability and the interaction did not affect the number of steps. The same pattern was found for the variability of time between steps. Here, the main effect of movement complexity was highly significant (*F*_1,19_ = 114.08, *p* < 0.001, *p*_crit_ = 0.05, ${\eta }_{p}^{2}\,$ = 0.86), with variability being higher for the obstacle course (*M* = 36.39, *SD* = 16.63) than for walking (*M* = 12.33, *SD* = 9.27; t_19_ = −10.68, *p* < 0.001, *p*_crit_ = 0.05). Considering the number of laps passed, no significant effect could be observed.

### Response times

**Figure 2:**
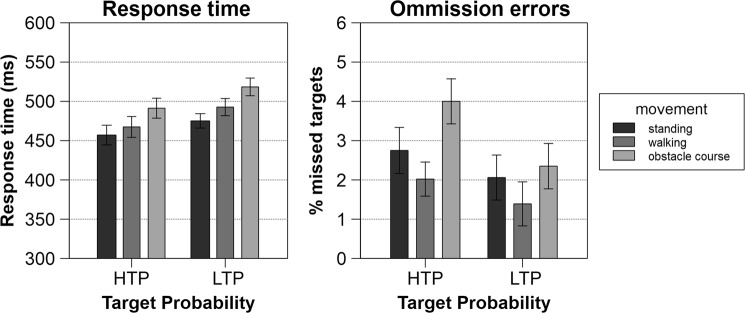
Response times and omission errors for every experimental condition. Error bars depict the standard error. HTP: high target probability (35%), LTP: low target probability (20%).

Figure 2 shows response times and mean accuracy for each of the six conditions. A main effect of target probability was found as response times for LTP (*M* = 495.42, *SD* = 64.71) were significantly slower than for HTP (*M* = 471.94, *SD* = 675.65; *F*_1,19_ = 5.68, *p* = 0.03, *p*_crit_ = 0.033, ${\eta }_{p}^{2}\,$ = 0.23). For the main effect of movement, response times were increased with increasing movement complexity (M_stand_ = 466.14, SD_stand_ = 55.33; M_walk_ = 480.06, SD_walk_ = 61.97; M_obstacle_ = 504.84, SD_obstacle_ = 61.62; *F*_2,38_ = 4.76, *p* = 0.02, *p*_crit_ = 0.05, ${\eta }_{p}^{2}$ = 0.20). Post-hoc tests revealed significant differences between standing and the obstacle course (t_38_ = −3.04, *p* = 0.004, *p*_crit_ = 0.05). When comparing means, a tendency towards slower response times in the walking as compared to the obstacle course condition emerges, but stays insignificant (t_38_ = −1.95, *p* = 0.058, *p*_crit_ = 0.033). The interaction of target percentage and movement complexity condition did not reach significance.

**Figure 3:**
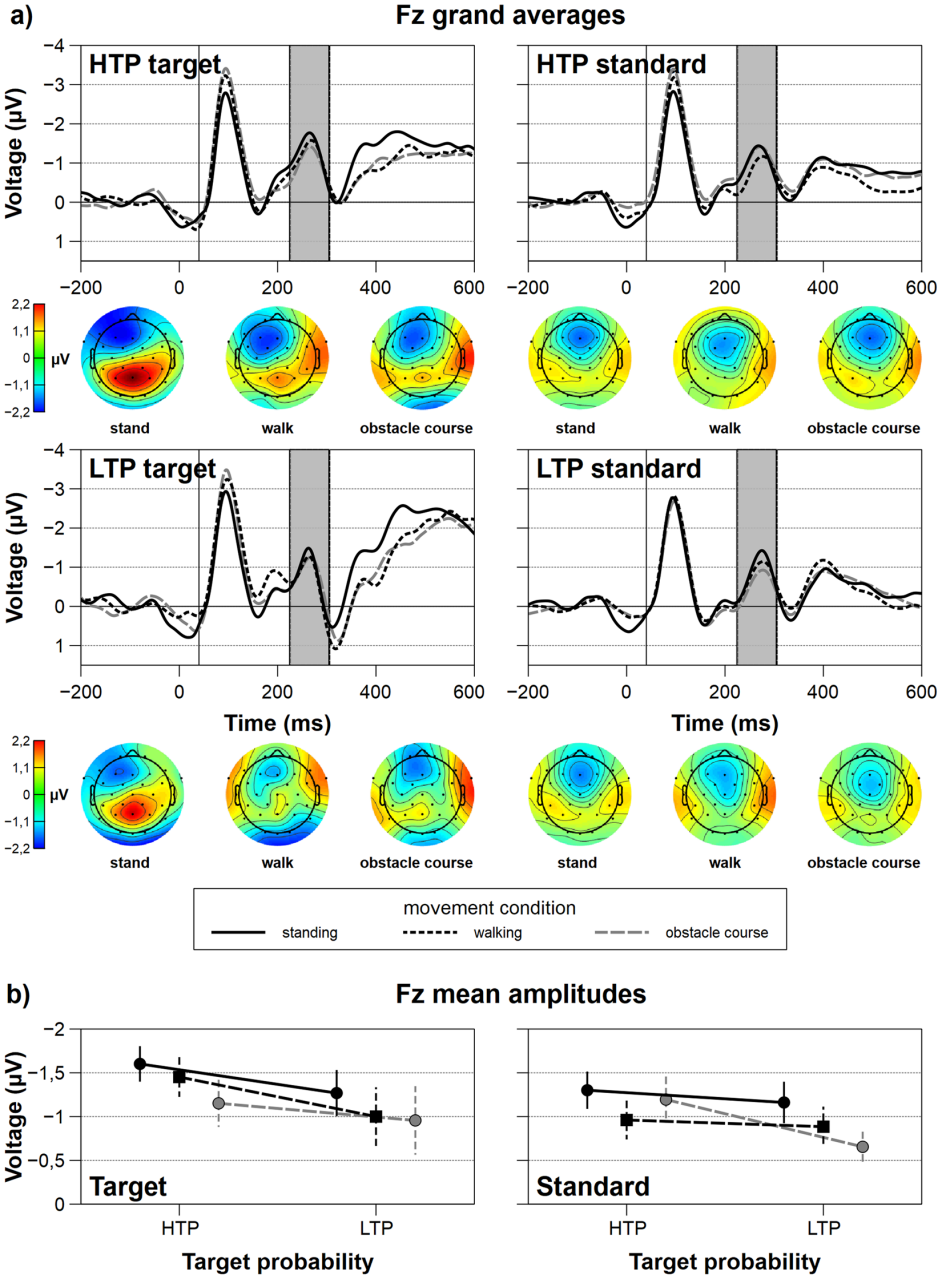
Frontal grand average ERPs in the N2 window and interaction plots for all factorial combinations. (**a**) Grand average N2 ERPs at Fz for all movement, target type and target probability conditions. The grey rectangles indicate the time windows for averaging. The topographies underneath the ERP-plots depict movement complexity specific voltages on the scalp in the N2 time-window. (**b**) Interaction plots for the N2 average amplitudes at Fz. Error bars depict the standard error. HTP: high target probability (35%), LTP: low target probability (20%).

**Figure 4:**
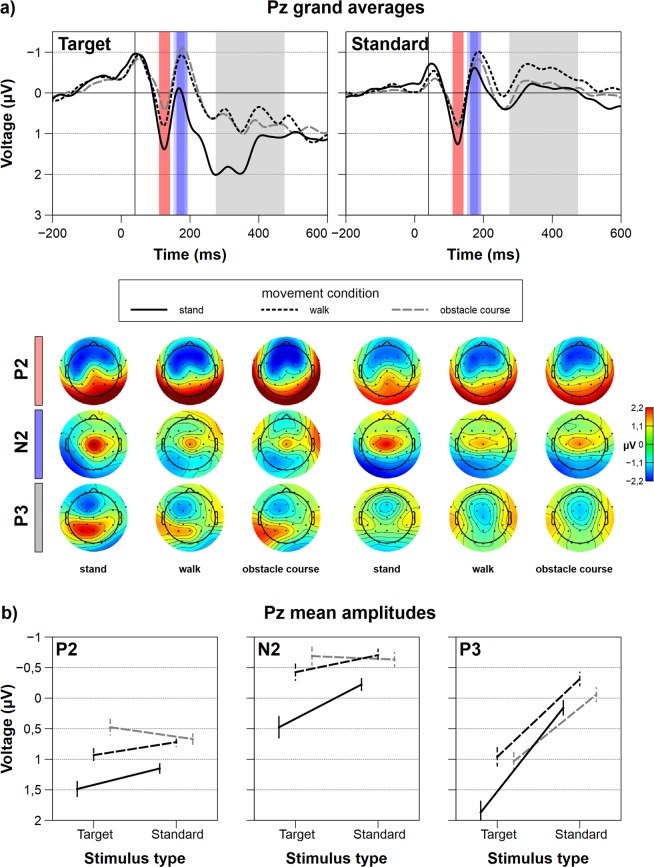
Parietal grand average ERPs in the P2, N2, and P3 window and interaction plots for all factorial combinations. (**a**) Grand average ERPs at Pz with time windows for the P2, N2 and P3 component for all movement and target type conditions. The colored rectangles indicate the time windows for averaging as follows: red – P2, blue – N2, grey – P3. Topographies for all component-specific time windows are provided underneath the ERPs. (**b**) Interaction plots for P2, N2, and P3 average amplitudes. The error bars depict the standard error.

**Figure 5:**
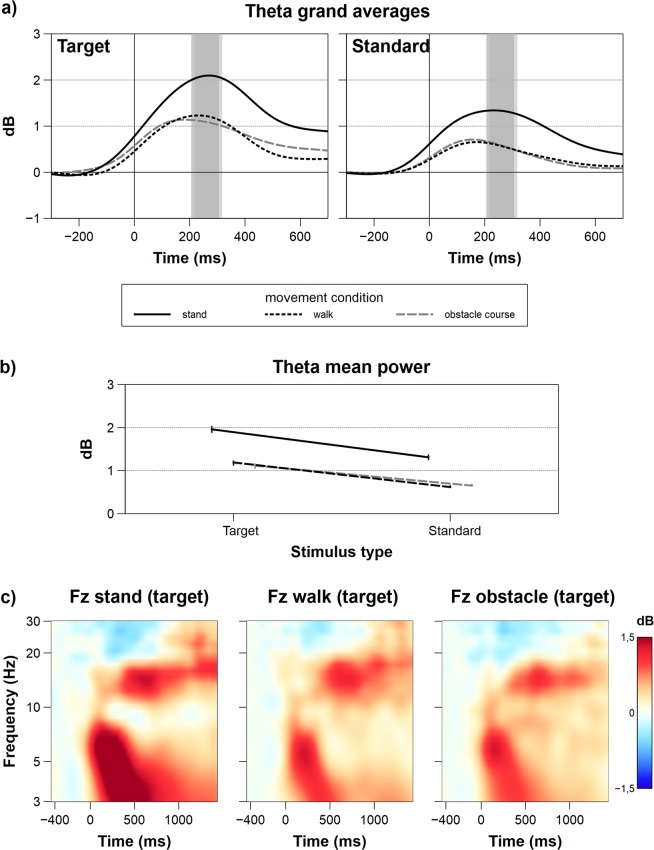
Graphs and interaction plots for frontal Theta power. (**a**) Grand average ERSP Theta power at Fz for all movement and target type conditions. The grey rectangles indicate the time windows for averaging. (**b**) Interaction plot for Theta average power. The error bars depict the standard error. (**c**) Time-frequency plots of target-only movement complexity conditions – not regarding target probability (TP) – for the frequency range between 3 and 30 Hz. Besides the effects in the Theta-range, one can see subtle distinctions in the Alpha- and low Beta-range for the movement complexity conditions.

Omission errors indicated similar results as only the main effect of movement (*F*_1,64,31.24_ = 3.70, p = 0.04, *p*_crit_ = 0.05, ${\eta }_{p}^{2}$ = 0.16) was significant. Due to the FDR-correction, target probability (*F*_1,19_ = 4.46, p = 0.05, *p*_crit_ = 0.03, ${\eta }_{p}^{2}$ = 0.19) turned out insignificant. Considering movement, the only significant pairwise post-hoc difference (t_38_ = −2.72, *p* = 0.01, *p*_crit_ = 0.05) emerged between walking (*M* = 0.017, *SD* = 0.025) and the obstacle course (*M* = 0.032, *SD* = 0.030).

### ERPs

#### Frontal N2 at Fz

The frontal N2 ERP component did not vary significantly with target stimulus or movement complexity condition. Significant amplitude differences could be found for the factor target probability (*F*_1,19_ = 4.39, *p* = 0.05, *p*_crit_ = 0.05, ${\eta }_{p}^{2}\,$ = 0.19). HTP stimuli (*M* = −1.28, *SD* = 1.42) elicited a larger N2 response compared to LTP stimuli (*M* = −0.99, *SD* = 1.68). ERPs at Fz, topographies at the given time windows, and interaction plots of the frontal N2 amplitudes are depicted in Fig. 3.

#### Parieto-temporal P3 at Pz

Regarding the P3 ERP component, the main effects for movement complexity (*F*_1,61,30.61_ = 15.22, *p* < 0.001, *p*_crit_ = 0.043, ${\eta }_{p}^{2}$ = 0.44) as well as for target stimulus (*F*_1,19_ = 37.25, *p* < 0.001, *p*_crit_ = 0.05, ${\eta }_{p}^{2}$ = 0.65) were significant. Target stimuli resulted in a more positive deflection than standard stimuli. Paired post-hoc comparisons for movement complexity revealed significantly larger P3 amplitudes for standing (*M* = 1.01, *SD* = 1.56) compared to walking (*M* = 0.32, *SD* = 1.26; *p* < 0.001) and for standing compared to the obstacle course (*M* = 0.49, *SD* = 1.18; *p* < 0.001). There was also a tendency towards larger P3 amplitudes in walking compared to the obstacle course that remained insignificant (*p* = 0.06). The interaction of movement complexity and target stimulus reached significance (*F*_1.87,35.54_ = 4.00, *p* = 0.03, *p*_crit_ = 0.036 ${\eta }_{p}^{2}\,$ = 0.19), so we calculated pairwise post-hoc comparisons to evaluate specific effects within conditions. Within the target stimuli, standing was statistically different than walking (t_74.38_ = 5.27, *p* < 0.001, *p*_crit_ = 0.04) and the obstacle course (t_74.38_ = 4.84, *p* < 0.001, *p*_crit_ = 0.037). Amplitudes were highest for standing (*M* = 1.87, *SD* = 1.07), followed by the obstacle course (*M* = 1.03, *SD* = 0.98) and walking (*M* = 0.96, *SD* = 1.04). Within standard stimuli movement complexity was the only significant main effect (*F*_2,38_ = 7.05, *p* = 0.008, ${\eta }_{p}^{2}$ = 0.27). Again, standing (*M* = 0.16, *SD* = 0.67) showed the most positive amplitude, followed by the obstacle course (*M* = −0.06, *SD* = 0.62) and walking (*M* = −0.31, *SD* = 0.58). Significant differences were only found for standing and walking (t_74.38_ = 2.73, *p* = 0.007, *p*_crit_ = 0.013). Figure 4 shows ERPs at Pz, topographies at the given time windows, and the interaction plots of parieto-temporal P2, N2, and P3 amplitudes.

### Frontal event-related spectral perturbations (ERSP) Theta at Fz

The midfrontal Theta response exhibited a significant main effect for the factor movement complexity (*F*_1.54,39.24_ = 16.99, *p* < 0.001, *p*_crit = _0.043,${\eta }_{p}^{2}$ = 0.47). Post-hoc tests revealed that the frontal Theta amplitude was larger in the standing condition (*M* = 1.63, *SD* = 1.00) compared to walking (*M* = 0.90, *SD* = 0.83; *t*_38_ = 4.98, *p* < 0.001, *p*_crit_ = 0.033) and the obstacle course (*M* = 0.88,*SD* = 0.78; *t*_38_ = 5.12, *p* < 0.001, *p*_crit_ = 0.05). The main effect of target stimulus was also significant (*F*_1,19_ = 27.91, *p* < 0.001, *p*_crit_ = 0.05, ${\eta }_{p}^{2}$ = 0.59), with target stimuli eliciting larger Theta responses than standard stimuli. A significant interaction was found for the factors target probability and target stimulus (*F*_1,19_ = 9.99, *p* = 0.005, *p*_crit_ = 0.036, ${\eta }_{p}^{2}\,$ = 0.34). By looking at specific interaction effects within target probability factor levels, further post-hoc pairwise comparisons demonstrated that the difference between target (*M* = 1.29, *SD* = 0.91) and standard stimuli (*M* = 0.89, *SD* = 0.76) was larger in LTP blocks (*t*_38_ = −6.14, *p* < 0.001, *p*_crit_ = 0.05) compared to target (*M* = 1.56, *SD* = 1.01) and standard stimuli (*M* = 0.83, *SD* = 0.68) in HTP blocks (*t*_38_ = −3.35, *p* = 0.002, *p*_crit_ = 0.025). Frontal ERSPs of Theta power at Fz, interaction plots of frontal theta power, and time-frequency plots at Fz are depicted in Fig. 5.

## Discussion

In this study, we used a mobile and lightweight EEG measurement setup and recorded subjective, behavioural, and electrophysiological measures of participants performing a cognitive-motor dual-task in an outdoors environment. While performing an auditory oddball task, the participants either stood, walked laps, or completed the laps with obstacle course elements. In contrast to previous studies on cognitive-motor interference dual-tasks^1,14,53^, we pursued an approach to investigate basic attentional mechanisms embedded in ecologically valid conditions. Recent advances in the technological development of recording equipment^9^ and stimulus presentation^54^ increased the feasibility of mobile experiments. Also, prior studies using mobile EEG approaches outside of the laboratory demonstrated that high ecological validity does not necessarily decrease signal quality^11–13,55^. Since the data of all experimental conditions were collected at the same location, we kept environmental factors like temperature, lighting or sensory complexity comparable.

With respect to the manipulation of movement complexity the results are clearly in line with our initial hypothesis. The behavioural measures indicate that a higher complexity of motor activity is accompanied by a decline in performance. Response latencies were significantly increased in the walking and the obstacle course condition as compared to the standing condition. There was also a tendency towards slower responses in the obstacle course compared to the walking condition. A similar pattern emerges for omission errors, since during the obstacle course participants missed significantly more targets than during the other movement complexity conditions. Keeping in mind that the motor task should be prioritized by the participants in order to reduce the risk of injury, this overall decrement in performance may indicate a decreased availability of attentional resources for cognitive performance with higher motor workload. This interpretation is also supported by the subjective ratings, as the participants evaluated the cognitive and physical demand, as well as general effort higher and also rated their own performance lower with increasing complexity of the motor conditions.

This cognitive-motor interference is clearly visible in the EEG as well. As hypothesized, the parietal P3 amplitude demonstrated a significant distinction between all movement complexity conditions. The P3 amplitude in response to target stimuli was significantly decreased in both locomotion conditions as compared to the standing condition. As the parietal P3 has been linked to the allocation of cognitive resources^38,44^, this observation further strengthens the interpretation of the reduced availability of attentional resources to be responsible for the decline of performance with increasing movement complexity. The analysis of frontal midline Theta power revealed comparable results. The event-related increase in Theta power was significantly decreased in the walking and the obstacle course condition compared to standing, indicating a decline in focused attention for mental task execution while in motion^47,56^. Also, it could be shown, that in conditions with higher frontal Theta power (standing), participants responded fastest. By this connection Theta power might reflect cognitive effort and could therefore allow for a connection between electrophysiology and resource allocation^46^. Also, when referring to the review of Roux and Uhlhaas^57^ about oscillations and working memory, there is a clear connection between working memory maintenance and Theta activation. Decreased Theta power during higher motor load could therefore indicate impaired working memory maintenance processes and account for the deteriorated response times. Altogether, regarding prior dual-task interference literature, these electrophysiological results suggest that both the motor and the cognitive task share a common pool of attentional resources^31,58^. This is in line with previous findings, demonstrating differences in resource allocation for non-motor and motor tasks^1,13,14,59^.

Interestingly, when looking only at standard stimuli, P3 amplitude was significantly negatively enhanced only for walking, possibly indicating cognitive processing of no-go stimuli. Our participants also exhibited a significantly smaller number of omission errors while walking as compared to the standing and obstacle course conditions. These results may in theory indicate a non-linear variation of task-difficulty from standing to walking to obstacle course, at least with respect to specific task-demands. The analysis of the gait-measures, however, does not support this interpretation. Participants took more steps and had a higher variability between steps during the obstacle course compared to normal walking, but completed a similar number of laps. Considering the review by Al-Yahya *et al*.^25^ these results might indicate a difference of overall workload induced by the movement complexity, especially since the obstacle elements forced a certain gait-pattern onto the participants, so that a significant difference in workload might be caused by correct task-execution itself.

The analysis of the experimental factor target probability partially suggested the replication of previous laboratory findings. Being in line with our hypothesis, subjective ratings indicated less cognitive effort and a better subjective performance for the LTP condition. Adversely, response times were slower for LTP stimuli, while participants made less omission errors. In contrast to our expectations as well as to previous findings^36,52^, the frontal Theta power and the parietal P3 amplitude were not modulated by the factor target probability. The N2 amplitude, however, revealed a main effect of target probability with larger negative deflections in the LTP compared to the HTP condition. Target-probability effects for the N2 have rarely been researched and adverse findings have been reported from the visual^34,60^ and auditory domain^61^. It may be that higher N2 amplitudes in the LTP condition reflect a greater need for cognitive control^41,62^ when there are less targets, which might also explain slower responses. We also observed a significant interaction of the factors target stimulus and target probability for frontal Theta power, reflecting higher Theta power for targets within LTP compared to HTP as hypothesized^52^. In summary, participants rated themselves to be better, had more negative N2 amplitudes and higher Theta power, but showed slower response times during the LTP conditions. It thus seems that a higher target probability does not influence cognitive workload, but seems to influence the cognitive system in a rather general manner. Thus, certain effects of target probability found in a laboratory setting were present in this study, main findings of target P3 amplitude variation could not be replicated.

Overall, the results clearly show that executing a well-known laboratory paradigm in a natural environment is feasible, which is in line with previous findings^12,14,55,59^. Besides replicating the oddball effect outdoors, the data provides new evidence on the topic of cognitive-motor interference. Firstly, a clear limitation of the presented approach is that the task is very basic and therefore lacks ecological validity. Secondly, presenting auditory stimuli over passive noise-cancelling headphones might induce additional cognitive demand, since perception of naturally occurring environmental stimuli and, consequentially, orientation in said natural environment in can be impaired. Due to the jittered stimulus presentation these additional cognitive demands should not induce systematic error variance though. Finally, to keep results comparable to prior literature, we ensured that external influences (temperature, lighting etc.) were similar during each conduction of the experiment which might restrict ecological validity further. Future research should address this limitation by investigating cognition in more complex and application-related scenarios. Nevertheless, it could be shown that a manipulation of movement complexity led to distinct effects in subjective, behavioural and electrophysiological measures. The fact that motor complexity and not speed was manipulated provides strong evidence that an increased motor load reduces the cognitive resources available for cognitive processes.

## Methods

### Participants

In this study 29 healthy subjects participated. All of the participants were free of prior or present neurologic or psychiatric conditions, right-handed, and had normal or corrected-to-normal vision. None of the participants reported hearing deficiencies. Due to problems concerning trigger generation in early stages of the experiment, only datasets of 20 participants (11 male, 9 female) could be used for further analysis. The subjects’ age in the concluding sample ranged from 20 to 30 years (*M* = 24.10, *SD* = 3.08). Subjects were paid 10 € per hour and gave their informed consent. The study was approved by the local ethics committee of the Leibniz Research Centre for Working Environment and Human Factors and was conducted in accordance with the Declaration of Helsinki.

### Apparatus and stimuli

**Figure 6:**
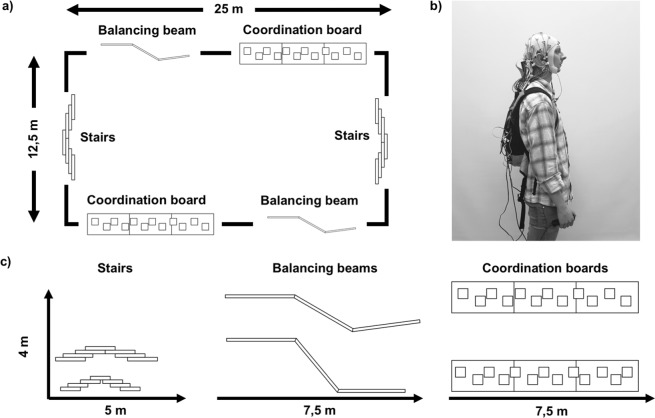
Depiction of (**a**) the obstacle course, (**b**) a participant with full experimental equipment, and (**c**) the elements of the obstacle course. (**a**) The obstacle course had a circumference of 75 m and consisted of two stairs, two balancing beams and two coordination boards. Participants had to climb and descend the stairs, balance over the beams without touching the adjacent ground and complete the coordination boards by placing their feet in the hole cut-outs without touching the surrounding surface. (**b**) The participant wore a 30-electrode cap, which was connected to a mobile amplifier placed in a pocket at the back of the head. In the backpack a raspberry Pi was stored which generated auditory stimuli and trigger signals. Triggers were transferred into digital signals via a Trigger extension box which then was connected to the amplifier. The participant held a handle with a reaction button in the right hand. By pressing the button with the right thumb, response triggers were inserted into the EEG-signal via the Trigger extension. EEG-data was stored offline on an SD-card inside the amplifier. (**c**) The staircases were 0.58 m high, had a length of 3.2 m/4.1 m, and a width of 1,2 m. Balancing beams were 0.06 m high and approximately 7,30 m long with angles between the planks reaching from 130° to 150°. The coordination boards were 7.5 m long with a height of 0.08 m. Hole cut-outs were 0.4 m by 0.4 m with 0.05 m up to 0.3 m of space in-between.

The whole data acquisition took place on the outside terrain of the institute between June 26^th^ and August 29^th^ 2018. The obstacle course used for implementing the most complex motor task consisted of two small staircases, two balancing beams, and two boards with punched-out holes (Fig. 6c). The course for the walking and the obstacle course condition had a circumference of 75 meters. The obstacle course was situated on the lawn on the outside premises of the institute, so subjects walked on a grass field with common surface irregularities during the walking or obstacle course conditions. The outside temperatures at the beginning of the experimental procedure ranged from 15 °C to 28 °C.

To be able to record EEG data in this natural setting, a mobile setup for stimulus generation and data acquisition was used. A Raspberry Pi 2B (Raspberry Foundation, UK) with custom OpenSesame Scripts^63^ was used for stimulus presentation and – in combination with the LiveAmp Sensor and Trigger Extension (Brain Products GmbH, Gilching, GER) – for trigger generation. To enable behavioural responses by the subject, a self-made handle with a response button was used which was connected to the Trigger Extension. The subjects were instructed to operate the handle with their right hand (Fig. 6b). The devices were stored in a small backpack which the participant wore for the duration of the experiment. The picture shows the first author of this manuscript during a pilot study. Written informed consent to publish this picture was obtained. In order to prevent induction currents due to cable sway^64^, all wires were prepared not to cross each other and were taped together for protection from movement.

Auditory stimuli were presented to the participants via passive noise-cancelling in-ear headphones (Bose QC25, Framingham, USA). The tones presented consisted of pure sine waves (low: 600 Hz, high: 900 Hz) with a length of 62 ms (10 ms rise/fall time). Stimuli were presented with an inter-stimulus interval of 2000 ms with a jitter of +/−250 ms.

### Procedure

Upon arrival between 8 and 9 am, the participants read the experiment information and signed the informed consent. Subsequently, they were fitted with a 30 electrode actiCap setup (actiCAP Slim electrodes & actiCAP Snap cap, Brain Products GmbH, Gilching, GER) as well as the backpack and were accompanied outside to the obstacle course track. For each participant the preparation took approximately 1 hour.

At the course the participants were fully instructed about the task execution. For the dual-task approach participants had to perform a cognitive and a motor task simultaneously. The cognitive task consisted of a simple signal-detection auditory oddball task. Participants were either presented with a low-pitched standard-tone (600 Hz) at an average sound level of approximately 65 dB(A) or with a high-pitched target-tone (900 Hz) at an average sound level of approximately 72 dB(A) in a randomized stream of stimuli. Sound levels were measured directly at the earphones’ earmold sound outlet. The participants were instructed to respond to a target tone by pressing the response button and to withhold a response when hearing a standard tone. The stream of stimuli presented to the participants consisted either of 80% standard and 20% target tones in the LTP condition or of 65% standard- and 35% target-tones in the HTP condition. For the motor task, participants had to either stand still on a predetermined spot (stand) in the lower left corner of the obstacle course, walk around the obstacle course (walk), or include the obstacles of the course while walking (obstacle course, see Fig. 6a). Whether a participant performed the motor task clockwise or anticlockwise was quasi-randomized and counterbalanced across participants. The sequence of tasks was quasi-randomized using a six factorial latin square design.

After each task, the participants’ subjective task-load was assessed via the NASA-TLX^37,65^ questionnaire. The participants also had the possibility for a short rest. For each factor combination of the 2 (target probability) × 3 (movement complexity) design the subjects were presented with 450 Stimuli. Each experimental block had a duration of 15 minutes, totalling to an overall duration of 1.5 hours. After finishing the task, the subjects were escorted back into the laboratory, where the electrode cap and the backpack were removed. Overall, the experimental procedure took roughly 3.5 to 4 hours per participant.

### Electrophysiological data acquisition

Since mobile and real-world EEG setups are quite new, this section will explain the EEG equipment and subject preparation in higher detail than usual^66^. EEG data was acquired via 30 active electrodes in a standard 10–20-system montage (Fp1, Fp2, F3, F4, F7, F8, Fz, FC1, FC2, FC5, FC6, C3, C4, Cz, T7, T8, CP1, CP2, CP5, CP6, P3, P4, P7, P8, Pz, PO9, PO10, O1, O2, Oz). To fully prepare a participant for recording, a tight fitting, flexible cap with electrode holders was pulled over the subject’s head at first. Sizes of caps ranged from 54 to 60 cm in circumference. Then, actively shielded electrodes were inserted into the electrode holders and filled with conductive gel until they reached an impedance of 10 kΩ or below. Because of the low weight, profile, and centre of gravity of the electrodes, mechanical electrode displacement was prevented to a considerable degree. Additionally, electrode cables were carefully aligned to not cross each other or sway around while the participant was moving. Therefore, all cables were routed through specific cable mounts of the actiCap next to the participant’s ears. To further reduce cable motion, we bundled cables from the left and the right side and taped them together respectively. These cables were then routed to the mobile amplifier which was placed in a pocket at the back of the participant’s head. Data were recorded with a LiveAmp amplifier (Brain Products GmbH, Gilching, GER) with a sampling frequency of 500 Hz and a bit depth of 24 bit. All data were stored on a micro SD card inside the amplifier while the online signal could be viewed via a Bluetooth connection on a windows laptop running the Brainvision recorder software (Brain Products GmbH, Gilching, GER). After the experiment the data from the SD card were transferred to the laptop’s hard drive with the help of the LiveAmp File Converter software (Brain Products GmbH, Gilching, GER). FCz was used as online reference and AFz served as the ground. Head movements were recorded simultaneously with the help of the LiveAmp’s built-in gyro sensors.

### Data processing

The recorded and converted EEG data were pre-processed offline using custom Matlab and EEGLab scripts^67^. Separate pre-processing parameters and routines were used for event-related potentials (ERP) and event-related spectral perturbations (ERSPs). In general, the pre-processing was handled similarly to lab-based experiments. Previous research showed that artefactual data is produced mainly because of mechanical cable displacement and cable crossing^64^, which we prevented by proper cable management and cable relief mechanisms. Also, strong displacement artefacts were formerly shown to be elicited only by exceptional movement intensity like in a running task^14^. Comparable data quality and P300 classification for indoor seated and outdoor movement conditions were reported by Debener *et al*.^13^ as early as 2012. Additionally, ERPs and ERSPs should not be prone to direct movement artefacts due to the averaging process. Only when a movement is locked to a specific point in time related to the task this should pose a problem to the validity of the outcome.

For ERPs, the data were bandpass-filtered between 0.5 and 20 Hz using a fourth-order IIR Butterworth filter with DC-offset removal. For ERSP analysis, data were bandpass-filtered between 0.5 and 30 Hz. Then, two statistical channel rejections were performed with the first excluding all channels with a kurtosis of 8 SD or higher and the second excluding all channels with a probability higher than 5 SDs. These channels were excluded from both the ERP and ERSP dataset. Then the data were average-referenced to the remaining electrodes. On average, 2,2 channels were excluded per subject.

In a next step, the ERP data were sampled down to 125 Hz and saved in a separate dataset solely for the purpose of independent component analysis (ICA). The downsampling was necessary to facilitate ICA computation. Before running the IC decomposition, data from the ERP and ICA dataset were segmented into epochs ranging from −0.3 to 1.2 seconds relative to the stimulus onset.

For the ERSP data, the signal was sampled down to 250 Hz and epochs were created ranging from −1s to 2 s regarding stimulus onset. The downsampling was executed due to data handling purposes and the facilitation of further computations.

With the help of an automated trial rejection (voltage threshold: 1000 µV, probability threshold: 5 SD, maximum percent of total trials to reject per iteration: 10%), artefactual trials were detected in the ERP dataset and subsequently deleted from all separate datasets (ERP, ICA, ERSP). After trial rejection, an extended ICA was performed on the ICA dataset.

Then, IC-weights were transferred back to the original ERP- and ERSP-data. To reduce individual bias in data cleaning, independent components (ICs) representing artefacts were detected via the ADJUST plugin^68^. These artefactual ICs were then deleted from the ERP and ERSP data. Subsequently, previously excluded EEG-channels were spherically interpolated.

#### Questionnaires and behavioural data

For subjective workload evaluation a German translation of the NASA-TLX was used. Raw scores without factor weighting were passed on for statistical evaluation for each of the six dimensions. Behavioural responses were calculated as the latency difference between the stimulus-onset and right-hand button press. Only correctly detected target trials were considered for subsequent analyses. A trial was counted as correct, if the participant pressed the button following a target stimulus. Further analyses regarding incorrect responses and omission errors were not pursued, given the small proportion of errors (max 2.7%).

Walking data was analysed using the amplifier’s gyro sensor data. For walking and the obstacle course conditions, after band-pass filtering between 0.1 Hz and 20 Hz, average stepping frequency was detected by identifying the power-peak of the gyro acceleration between 0.5 Hz and 3 Hz. Then the original gyro data was filtered using a wavelet at this peak-frequency. At last, positive peaks of this filtered signal were used as step-markers for calculating number of steps and step interval variability.

#### EEG signal processing and analysis

ERPs: Using the EEGLab study design, correct standard and target trials were averaged for the six conditions across subjects. The baseline interval ranged from −200 ms to 0 ms pre-stimulus. For statistical analysis frontal N2- as well as parietal P2-, N2-, and P3-voltages were quantified as averages in specific time windows. To obtain specific frontal N2 and parietal P2 and N2 time-windows, grand-averages were calculated for each movement complexity condition’s target trials irrespective of target probability. By detecting the maximum voltage for these grand average ERPs frontal N2 between 150 ms and 300 ms relative to stimulus onset, the N2 time window was determined by placing a 50 ms window around the peaks (stand: 264 ms +/−25 ms, walk: 266 ms +/−25, obstacle course: 266 ms +/−25 ms). The N2 was then parameterized as the mean amplitude in these time windows for each factor combination. The same procedure was performed for parietal P2 (search between 100 ms and 200 ms) and N2 (search between 150 ms and 300 ms) to obtain grand average peaks. Distinct peaks were found for both the parietal P2 (stand: 126 ms, walk: 124 ms, obstacle course: 126 ms) and N2 (stand: 168 ms, walk: 176 ms, obstacle course: 178 ms). A time window of +/−20 ms was applied around the peaks to parametrize parietal P2 and N2 mean voltages. Due to the lack of temporal specificity, the P3 amplitude measure was parameterized as the mean amplitude in the time window ranging from 250 ms to 500 ms relative to the stimulus onset.

ERSPs: Similarly to ERP measures, only correct standard and target trials were used for ERSP computation. ERSP data was calculated using an EEGLab study. 28 Wavelets with three cycles and a cycle shortening factor of 0.5 were computed for frequencies between 3 Hz and 30 Hz. The baseline was set for the interval between −500 ms and −200 ms relative to stimulus onset. Theta power was averaged between 4 and 7 Hz before determining the movement complexity conditions’ peak latencies analogously to the N2. The determined time-windows were 268 ms +/−150 ms for the standing condition, 256 ms +/−150 ms for the walking condition and 256 ms +/−150 ms for the obstacle course condition. Theta power was then averaged in these time windows for each factor combination.

### Statistical analysis

Statistical analyses were carried out using R version 3.4.3^69^ and the ez-package^70^. For subjective and behavioural data 2 × 3 repeated measures analyses of variance (ANOVAs) were calculated using movement complexity condition and target probability as within factors. Regarding physiological measures, we used 2 × 3 × 2 repeated measures ANOVAs with movement complexity condition, target probability, and stimulus type (target/non-target) as within factors. For the testing of statistical significance, p-values equal to or below 0.05 were considered as significant. To account for family-wise error accumulation, significances within each ANOVA were corrected for false discovery rate (FDR) as indicated by Cramer and colleagues^71^. Post-hoc tests for movement complexity conditions were calculated using pairwise paired t-test comparisons for all factorial levels. Post-hoc test probabilities were also corrected for FDR. In cases of FDR-correction, adjusted critical p—values (p_crit_) are provided. The ANOVAS’ effect sizes are reported in ${\eta }_{p}^{2}$. Statistical plots and figures with a significant main effect of target probability display all possible factors; for measures without a significant TP main effect, plots were produced without splitting the graphs into TP-levels to facilitate comprehensibility and to declutter the plots.

## Data Availability

The datasets generated during and/or analysed during the current study are available from the corresponding author on reasonable request.
